# (Acetato-κ^2^ *O*,*O*′)(acetato-κ*O*)bis(2-amino-3-methyl­pyridine-κ*N* ^1^)cobalt(II)

**DOI:** 10.1107/S1600536812038664

**Published:** 2012-09-15

**Authors:** Azadeh Tadjarodi, Keyvan Bijanzad, Behrouz Notash

**Affiliations:** a Department of Chemistry, Iran University of Science and Technology, Tehran 16846-13114, Iran; b Department of Chemistry, Shahid Beheshti University, G. C., Evin, Tehran 1983963113, Iran

## Abstract

In the title compound, [Co(CH_3_COO)_2_(C_6_H_8_N_2_)_2_], the Co^II^ ion is five-coordinated by two pyridine N atoms from two 2-amino-3-methyl­pyridine ligands, two O atoms from one acetate ion and one O atom from another acetate ion in a distorted trigonal–bipyramidal geometry. The pyridine rings are nearly perpendicular to each other [dihedral angle = 84.49 (16)°]. The crystal packing is stabilized by intra­molecular and inter­molecular N—H⋯O hydrogen-bonding inter­actions.

## Related literature

For related coordination compounds of 2-amino-3-methyl­pyridine, see: Arab Ahmadi *et al.* (2011 ▶); Tadjarodi *et al.* (2010 ▶, 2012 ▶); Castillo *et al.* (2001 ▶); Ziegler *et al.* (2000 ▶); Amani Komaei *et al.* (1999 ▶); Chen *et al.* (2005 ▶). For proton-transfer compounds of 2-amino-3-methyl­pyridine, see: Carnevale *et al.* (2010 ▶).
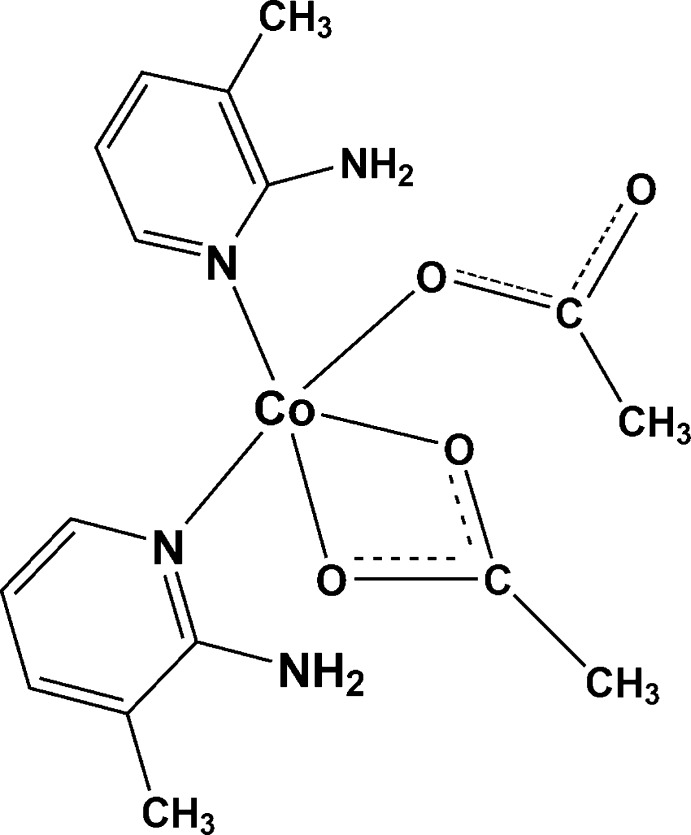


## Experimental

### 

#### Crystal data

[Co(C_2_H_3_O_2_)_2_(C_6_H_8_N_2_)_2_]
*M* *_r_* = 393.31
Triclinic, 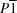

*a* = 8.1685 (16) Å
*b* = 10.452 (2) Å
*c* = 12.231 (2) Å
α = 69.58 (3)°
β = 79.94 (3)°
γ = 72.42 (3)°
*V* = 930.1 (4) Å^3^
*Z* = 2
Mo *K*α radiation
μ = 0.95 mm^−1^
*T* = 298 K
0.27 × 0.23 × 0.13 mm

#### Data collection

Stoe IPDS 2T diffractometer
Absorption correction: numerical (*X-SHAPE* and *X-RED32*; Stoe & Cie, 2005 ▶) *T*_min_ = 0.785, *T*_max_ = 0.886
11215 measured reflections
4996 independent reflections
2756 reflections with *I* > 2σ(*I*)
*R*_int_ = 0.062

#### Refinement

*R*[*F* ^2^ > 2σ(*F* ^2^)] = 0.051
*wR*(*F* ^2^) = 0.096
*S* = 0.92
4996 reflections
242 parameters
3 restraints
H atoms treated by a mixture of independent and constrained refinement
Δρ_max_ = 0.35 e Å^−3^
Δρ_min_ = −0.19 e Å^−3^

### 

Data collection: *X-AREA* (Stoe & Cie, 2005 ▶); cell refinement: *X-AREA*; data reduction: *X-AREA*; program(s) used to solve structure: *SHELXS97* (Sheldrick, 2008 ▶); program(s) used to refine structure: *SHELXL97* (Sheldrick, 2008 ▶); molecular graphics: *ORTEP-3 for Windows* (Farrugia, 1997 ▶); software used to prepare material for publication: *WinGX* (Farrugia, 1999 ▶).

## Supplementary Material

Crystal structure: contains datablock(s) I, global. DOI: 10.1107/S1600536812038664/xu5616sup1.cif

Structure factors: contains datablock(s) I. DOI: 10.1107/S1600536812038664/xu5616Isup2.hkl

Additional supplementary materials:  crystallographic information; 3D view; checkCIF report

## Figures and Tables

**Table 1:** Selected bond lengths (Å)

Co1—O1	1.962 (2)
Co1—O3	2.352 (2)
Co1—O4	2.0028 (18)
Co1—N1	2.072 (2)
Co1—N3	2.074 (2)

**Table 2:** Hydrogen-bond geometry (Å, °)

*D*—H⋯*A*	*D*—H	H⋯*A*	*D*⋯*A*	*D*—H⋯*A*
N2—H2*A*⋯O4	0.85 (2)	2.17 (2)	2.965 (3)	157 (3)
N2—H2*B*⋯O2^i^	0.84 (2)	2.16 (2)	2.978 (3)	166 (3)
N4—H4*A*⋯O1	0.83 (3)	2.10 (3)	2.859 (3)	153 (3)
N4—H4*B*⋯O3^ii^	0.84 (2)	2.06 (2)	2.881 (3)	164 (3)

Symmetry codes: (i) 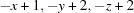; (ii) 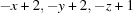.

**Fig. 1.**
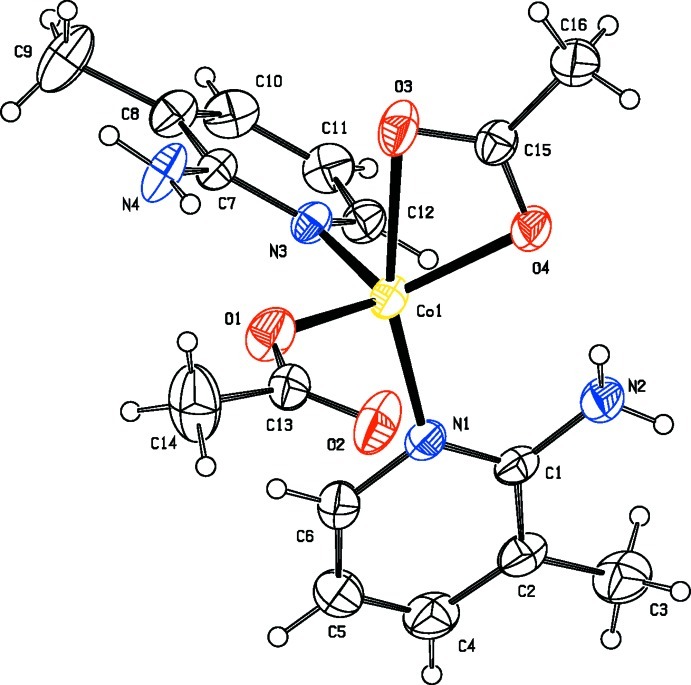
The molecular structure of [Co(ampy)2(CH3COO)2] with displacement ellipsoids drawn at 30% probability level.

**Fig. 2.**
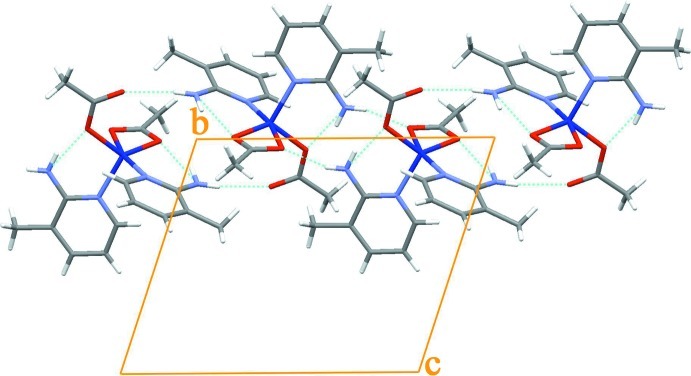
The packing diagram of the title compound showing hydrogen bonding as blue dashed lines.

## supplementary crystallographic information

### Comment

2-Amino-3-methylpyridine (ampy) coordinates to metals mostly through the nitrogen atom of the pyridyl group (Arab Ahmadi *et al.*, 2011; Tadjarodi *et al.*, 2012 and 2010; Castillo *et al.*, 2001; Ziegler *et al.*, 2000; Amani Komaei *et al.*, 1999) but it can also coordinate *via* the nitrogen atom of the amino group (Chen *et al.*, 2005). In recent years, several structures of proton-transfer compounds, [(ampyH)_2_Co*X*_4_] (*X* = Cl, Br) have been reported by 2-Amino-3-methylpyridine (Carnevale *et al.* 2010).

Herein, we report the synthesis and structural determination of the title compound, [Co(ampy)_2_(CH_3_COO)_2_]. The coordination sphere of the mononuclear complex includes three oxygen atoms from two acetate ions and two pyridyl nitrogen atoms from two ampy ligands thus constructing a distorted trigonal bipyramidal geometry (Fig. 1). In the structure of [Co(ampy)_2_(CH_3_COO)_2_], several intramolecular and intermolecular N–H···O hydrogen bond interactions formed between the amino group of the ligand and the acetate oxygen atoms which can stabilize the crystal structure (Fig. 2 & Table 1).

### Experimental

A solution of 2-amino-3-methylpyridine (1 mmol) in ethanol was added to an aqueous solution of Co(CH_3_COO)_2_.4H_2_O (0.5 mmol) and stirred for 20 min at 50 °C. Slow evaporation of the resulting solution gave violet plate shaped crystals of the title compound suitable for X-ray analysis (decomposition >300 °C).

### Refinement

Hydrogen atoms attached to nitrogen atoms were found in difference Fourier map.H2A and H2B and H4B were refined with distance restraints of N—H 0.845 (18), 0.840 (18) and 0.839 (18), respectively. H atoms attached to carbon atoms were positioned geometrically and refined as riding atoms, with C—H = 0.93 Å (CH), with C—H = 0.96 Å (CH_3_), and *U*_iso_(H) = 1.2,1.5*U*_eq_(C).

### Crystal data

**Table:**

[Co(C_2_H_3_O_2_)_2_(C_6_H_8_N_2_)_2_]	*Z* = 2
*M**_r_* = 393.31	*F*(000) = 410
Triclinic, *P*1	*D*_x_ = 1.404 Mg m^−^^3^
Hall symbol: -P 1	Mo *K*α radiation, λ = 0.71073 Å
*a* = 8.1685 (16) Å	Cell parameters from 4996 reflections
*b* = 10.452 (2) Å	θ = 2.2–29.2°
*c* = 12.231 (2) Å	µ = 0.95 mm^−^^1^
α = 69.58 (3)°	*T* = 298 K
β = 79.94 (3)°	Plate, violet
γ = 72.42 (3)°	0.27 × 0.23 × 0.13 mm
*V* = 930.1 (4) Å^3^	

### Data collection

**Table:**

Stoe IPDS 2T diffractometer	4996 independent reflections
Radiation source: fine-focus sealed tube	2756 reflections with *I* > 2σ(*I*)
Graphite monochromator	*R*_int_ = 0.062
rotation method scans	θ_max_ = 29.2°, θ_min_ = 2.2°
Absorption correction: numerical (*X-SHAPE* and *X-RED32*; Stoe & Cie, 2005)	*h* = −11→11
*T*_min_ = 0.785, *T*_max_ = 0.886	*k* = −14→14
11215 measured reflections	*l* = −16→16

### Refinement

**Table:**

Refinement on *F*^2^	Primary atom site location: structure-invariant direct methods
Least-squares matrix: full	Secondary atom site location: difference Fourier map
*R*[*F*^2^ > 2σ(*F*^2^)] = 0.051	Hydrogen site location: inferred from neighbouring sites
*wR*(*F*^2^) = 0.096	H atoms treated by a mixture of independent and constrained refinement
*S* = 0.92	*w* = 1/[σ^2^(*F*_o_^2^) + (0.0361*P*)^2^] where *P* = (*F*_o_^2^ + 2*F*_c_^2^)/3
4996 reflections	(Δ/σ)_max_ = 0.001
242 parameters	Δρ_max_ = 0.35 e Å^−^^3^
3 restraints	Δρ_min_ = −0.19 e Å^−^^3^

### Special details

**Table:**

Geometry. All e.s.d.'s (except the e.s.d. in the dihedral angle between two l.s. planes) are estimated using the full covariance matrix. The cell e.s.d.'s are taken into account individually in the estimation of e.s.d.'s in distances, angles and torsion angles; correlations between e.s.d.'s in cell parameters are only used when they are defined by crystal symmetry. An approximate (isotropic) treatment of cell e.s.d.'s is used for estimating e.s.d.'s involving l.s. planes.
Refinement. Refinement of *F*^2^ against ALL reflections. The weighted *R*-factor *wR* and goodness of fit *S* are based on *F*^2^, conventional *R*-factors *R* are based on *F*, with *F* set to zero for negative *F*^2^. The threshold expression of *F*^2^ > σ(*F*^2^) is used only for calculating *R*-factors(gt) *etc*. and is not relevant to the choice of reflections for refinement. *R*-factors based on *F*^2^ are statistically about twice as large as those based on *F*, and *R*- factors based on ALL data will be even larger.

### Fractional atomic coordinates and isotropic or equivalent isotropic displacement parameters (Å^2^)

**Table:**

	*x*	*y*	*z*	*U*_iso_*/*U*_eq_	
Co1	0.71818 (5)	0.93397 (4)	0.77010 (3)	0.04555 (13)	
O1	0.6158 (3)	1.0480 (2)	0.62129 (18)	0.0698 (6)	
O2	0.4417 (3)	1.2043 (3)	0.6980 (2)	0.0879 (8)	
O3	0.9471 (3)	1.0418 (2)	0.70296 (17)	0.0653 (6)	
O4	0.8186 (2)	0.9932 (2)	0.87698 (16)	0.0593 (5)	
N1	0.5289 (3)	0.8404 (3)	0.8723 (2)	0.0516 (6)	
N2	0.6160 (3)	0.8098 (3)	1.0513 (2)	0.0569 (6)	
N3	0.8801 (3)	0.7557 (2)	0.73364 (18)	0.0431 (5)	
N4	0.9101 (4)	0.8685 (3)	0.5353 (2)	0.0638 (7)	
C1	0.5153 (3)	0.7826 (3)	0.9897 (2)	0.0458 (6)	
C2	0.4051 (4)	0.6933 (3)	1.0468 (3)	0.0570 (8)	
C3	0.3999 (5)	0.6244 (4)	1.1759 (3)	0.0808 (11)	
H3A	0.3216	0.5655	1.1991	0.121*	
H3B	0.3618	0.6957	1.2141	0.121*	
H3C	0.5130	0.5676	1.1978	0.121*	
C4	0.3094 (4)	0.6719 (4)	0.9766 (3)	0.0737 (10)	
H4	0.2364	0.6131	1.0110	0.088*	
C5	0.3170 (4)	0.7344 (4)	0.8568 (3)	0.0813 (11)	
H5	0.2486	0.7206	0.8108	0.098*	
C6	0.4280 (4)	0.8170 (4)	0.8083 (3)	0.0684 (9)	
H6	0.4350	0.8593	0.7276	0.082*	
C7	0.9548 (3)	0.7526 (3)	0.6262 (2)	0.0435 (6)	
C8	1.0746 (4)	0.6286 (3)	0.6109 (3)	0.0540 (7)	
C9	1.1499 (5)	0.6286 (4)	0.4888 (3)	0.0899 (12)	
H9A	1.2360	0.5413	0.4930	0.135*	
H9B	1.2013	0.7063	0.4530	0.135*	
H9C	1.0602	0.6381	0.4430	0.135*	
C10	1.1139 (4)	0.5139 (3)	0.7075 (3)	0.0636 (8)	
H10	1.1933	0.4319	0.6997	0.076*	
C11	1.0377 (4)	0.5169 (3)	0.8177 (3)	0.0607 (8)	
H11	1.0649	0.4385	0.8837	0.073*	
C12	0.9222 (4)	0.6381 (3)	0.8255 (2)	0.0521 (7)	
H12	0.8689	0.6400	0.8988	0.063*	
C13	0.5012 (4)	1.1626 (3)	0.6146 (3)	0.0553 (7)	
C14	0.4425 (6)	1.2476 (5)	0.4946 (4)	0.1086 (15)	
H14A	0.3280	1.3066	0.5011	0.163*	
H14B	0.4430	1.1849	0.4528	0.163*	
H14C	0.5191	1.3057	0.4530	0.163*	
C15	0.9323 (4)	1.0452 (3)	0.8040 (2)	0.0492 (7)	
C16	1.0438 (4)	1.1079 (4)	0.8434 (3)	0.0724 (10)	
H16A	1.1267	1.0337	0.8925	0.109*	
H16B	0.9738	1.1690	0.8865	0.109*	
H16C	1.1027	1.1615	0.7763	0.109*	
H2A	0.666 (4)	0.874 (3)	1.016 (3)	0.087*	
H4A	0.842 (4)	0.941 (4)	0.545 (3)	0.087*	
H2B	0.587 (4)	0.798 (4)	1.1228 (17)	0.087*	
H4B	0.960 (4)	0.877 (4)	0.4681 (19)	0.087*	

### Atomic displacement parameters (Å^2^)

**Table:**

	*U*^11^	*U*^22^	*U*^33^	*U*^12^	*U*^13^	*U*^23^
Co1	0.0517 (2)	0.0480 (2)	0.0384 (2)	−0.01574 (17)	0.00583 (15)	−0.01758 (17)
O1	0.0657 (14)	0.0686 (15)	0.0551 (14)	0.0066 (12)	−0.0049 (10)	−0.0157 (12)
O2	0.1122 (19)	0.0934 (19)	0.0651 (16)	−0.0283 (15)	0.0210 (13)	−0.0454 (15)
O3	0.0945 (15)	0.0705 (15)	0.0374 (12)	−0.0391 (12)	0.0109 (10)	−0.0177 (10)
O4	0.0669 (13)	0.0814 (15)	0.0415 (11)	−0.0407 (11)	0.0106 (9)	−0.0227 (11)
N1	0.0485 (13)	0.0653 (16)	0.0505 (15)	−0.0228 (12)	0.0103 (10)	−0.0300 (13)
N2	0.0629 (16)	0.0674 (18)	0.0432 (15)	−0.0304 (13)	0.0048 (12)	−0.0134 (14)
N3	0.0491 (13)	0.0397 (13)	0.0371 (13)	−0.0156 (10)	0.0050 (10)	−0.0088 (11)
N4	0.090 (2)	0.0453 (16)	0.0374 (15)	−0.0059 (14)	0.0174 (13)	−0.0117 (13)
C1	0.0416 (14)	0.0437 (16)	0.0521 (17)	−0.0121 (12)	0.0104 (12)	−0.0209 (14)
C2	0.0567 (17)	0.0534 (19)	0.062 (2)	−0.0219 (14)	0.0157 (15)	−0.0234 (16)
C3	0.088 (3)	0.077 (3)	0.076 (3)	−0.046 (2)	0.013 (2)	−0.012 (2)
C4	0.073 (2)	0.076 (2)	0.089 (3)	−0.0447 (19)	0.0195 (19)	−0.037 (2)
C5	0.078 (2)	0.112 (3)	0.086 (3)	−0.056 (2)	0.0105 (19)	−0.052 (3)
C6	0.070 (2)	0.096 (3)	0.058 (2)	−0.0366 (19)	0.0070 (16)	−0.039 (2)
C7	0.0474 (15)	0.0409 (16)	0.0431 (16)	−0.0148 (12)	0.0059 (12)	−0.0161 (14)
C8	0.0600 (18)	0.0420 (17)	0.0564 (19)	−0.0130 (14)	0.0082 (14)	−0.0174 (15)
C9	0.116 (3)	0.061 (2)	0.072 (2)	−0.004 (2)	0.030 (2)	−0.030 (2)
C10	0.0599 (19)	0.0438 (18)	0.079 (2)	−0.0070 (15)	0.0005 (17)	−0.0184 (18)
C11	0.0646 (19)	0.0485 (19)	0.057 (2)	−0.0119 (15)	−0.0061 (15)	−0.0034 (15)
C12	0.0559 (17)	0.0555 (19)	0.0431 (17)	−0.0218 (15)	0.0030 (13)	−0.0103 (15)
C13	0.0556 (18)	0.062 (2)	0.0513 (18)	−0.0127 (16)	−0.0026 (14)	−0.0246 (16)
C14	0.135 (4)	0.091 (3)	0.082 (3)	0.021 (3)	−0.048 (3)	−0.030 (2)
C15	0.0570 (17)	0.0450 (17)	0.0443 (17)	−0.0170 (14)	0.0021 (13)	−0.0124 (13)
C16	0.073 (2)	0.093 (3)	0.068 (2)	−0.045 (2)	0.0090 (17)	−0.032 (2)

### Geometric parameters (Å, º)

**Table:**

Co1—O1	1.962 (2)	C4—C5	1.380 (5)
Co1—O3	2.352 (2)	C4—H4	0.9300
Co1—O4	2.0028 (18)	C5—C6	1.359 (4)
Co1—N1	2.072 (2)	C5—H5	0.9300
Co1—N3	2.074 (2)	C6—H6	0.9300
O1—C13	1.265 (4)	C7—C8	1.419 (4)
O2—C13	1.215 (3)	C8—C10	1.360 (4)
O3—C15	1.233 (3)	C8—C9	1.512 (4)
O4—C15	1.277 (3)	C9—H9A	0.9600
N1—C1	1.349 (3)	C9—H9B	0.9600
N1—C6	1.353 (3)	C9—H9C	0.9600
N2—C1	1.354 (4)	C10—C11	1.389 (4)
N2—H2A	0.845 (18)	C10—H10	0.9300
N2—H2B	0.840 (18)	C11—C12	1.354 (4)
N3—C12	1.345 (4)	C11—H11	0.9300
N3—C7	1.355 (3)	C12—H12	0.9300
N4—C7	1.331 (4)	C13—C14	1.501 (5)
N4—H4A	0.83 (3)	C14—H14A	0.9600
N4—H4B	0.839 (18)	C14—H14B	0.9600
C1—C2	1.414 (4)	C14—H14C	0.9600
C2—C4	1.367 (4)	C15—C16	1.492 (4)
C2—C3	1.489 (4)	C16—H16A	0.9600
C3—H3A	0.9600	C16—H16B	0.9600
C3—H3B	0.9600	C16—H16C	0.9600
C3—H3C	0.9600		
			
O1—Co1—O4	129.30 (10)	N1—C6—C5	122.9 (3)
O1—Co1—N1	104.36 (10)	N1—C6—H6	118.5
O4—Co1—N1	105.67 (8)	C5—C6—H6	118.5
O1—Co1—N3	103.15 (9)	N4—C7—N3	118.0 (2)
O4—Co1—N3	112.27 (9)	N4—C7—C8	121.0 (2)
N1—Co1—N3	97.38 (9)	N3—C7—C8	121.0 (3)
O1—Co1—O3	88.36 (9)	C10—C8—C7	117.8 (3)
O4—Co1—O3	58.90 (7)	C10—C8—C9	123.1 (3)
N1—Co1—O3	164.49 (7)	C7—C8—C9	119.1 (3)
N3—Co1—O3	88.12 (8)	C8—C9—H9A	109.5
C13—O1—Co1	119.87 (19)	C8—C9—H9B	109.5
C15—O3—Co1	83.56 (16)	H9A—C9—H9B	109.5
C15—O4—Co1	98.54 (16)	C8—C9—H9C	109.5
C1—N1—C6	118.6 (2)	H9A—C9—H9C	109.5
C1—N1—Co1	127.67 (17)	H9B—C9—H9C	109.5
C6—N1—Co1	112.7 (2)	C8—C10—C11	121.3 (3)
C1—N2—H2A	118 (2)	C8—C10—H10	119.4
C1—N2—H2B	118 (2)	C11—C10—H10	119.4
H2A—N2—H2B	116 (3)	C12—C11—C10	117.7 (3)
C12—N3—C7	118.3 (2)	C12—C11—H11	121.2
C12—N3—Co1	116.71 (17)	C10—C11—H11	121.2
C7—N3—Co1	124.82 (18)	N3—C12—C11	123.9 (3)
C7—N4—H4A	120 (3)	N3—C12—H12	118.0
C7—N4—H4B	123 (3)	C11—C12—H12	118.0
H4A—N4—H4B	116 (4)	O2—C13—O1	123.5 (3)
N1—C1—N2	117.2 (2)	O2—C13—C14	121.0 (3)
N1—C1—C2	121.9 (2)	O1—C13—C14	115.5 (3)
N2—C1—C2	120.9 (3)	C13—C14—H14A	109.5
C4—C2—C1	116.4 (3)	C13—C14—H14B	109.5
C4—C2—C3	122.9 (3)	H14A—C14—H14B	109.5
C1—C2—C3	120.8 (3)	C13—C14—H14C	109.5
C2—C3—H3A	109.5	H14A—C14—H14C	109.5
C2—C3—H3B	109.5	H14B—C14—H14C	109.5
H3A—C3—H3B	109.5	O3—C15—O4	119.0 (2)
C2—C3—H3C	109.5	O3—C15—C16	121.8 (3)
H3A—C3—H3C	109.5	O4—C15—C16	119.2 (2)
H3B—C3—H3C	109.5	C15—C16—H16A	109.5
C2—C4—C5	122.5 (3)	C15—C16—H16B	109.5
C2—C4—H4	118.7	H16A—C16—H16B	109.5
C5—C4—H4	118.7	C15—C16—H16C	109.5
C6—C5—C4	117.6 (3)	H16A—C16—H16C	109.5
C6—C5—H5	121.2	H16B—C16—H16C	109.5
C4—C5—H5	121.2		
			
O4—Co1—O1—C13	−50.1 (3)	Co1—N1—C1—C2	−164.79 (19)
N1—Co1—O1—C13	74.6 (2)	N1—C1—C2—C4	−1.8 (4)
N3—Co1—O1—C13	175.9 (2)	N2—C1—C2—C4	−179.6 (3)
O3—Co1—O1—C13	−96.4 (2)	N1—C1—C2—C3	176.5 (3)
O1—Co1—O3—C15	138.61 (18)	N2—C1—C2—C3	−1.3 (4)
O4—Co1—O3—C15	−0.62 (17)	C1—C2—C4—C5	−0.6 (5)
N1—Co1—O3—C15	−6.9 (4)	C3—C2—C4—C5	−179.0 (3)
N3—Co1—O3—C15	−118.18 (18)	C2—C4—C5—C6	1.7 (6)
O1—Co1—O4—C15	−56.9 (2)	C1—N1—C6—C5	−1.9 (5)
N1—Co1—O4—C15	178.84 (17)	Co1—N1—C6—C5	167.7 (3)
N3—Co1—O4—C15	73.83 (19)	C4—C5—C6—N1	−0.4 (5)
O3—Co1—O4—C15	0.60 (16)	C12—N3—C7—N4	178.7 (2)
O1—Co1—N1—C1	−161.2 (2)	Co1—N3—C7—N4	−6.2 (3)
O4—Co1—N1—C1	−22.5 (2)	C12—N3—C7—C8	−0.1 (4)
N3—Co1—N1—C1	93.1 (2)	Co1—N3—C7—C8	174.98 (19)
O3—Co1—N1—C1	−16.9 (5)	N4—C7—C8—C10	−179.8 (3)
O1—Co1—N1—C6	30.3 (2)	N3—C7—C8—C10	−1.0 (4)
O4—Co1—N1—C6	169.0 (2)	N4—C7—C8—C9	−0.9 (4)
N3—Co1—N1—C6	−75.3 (2)	N3—C7—C8—C9	177.9 (3)
O3—Co1—N1—C6	174.6 (3)	C7—C8—C10—C11	0.9 (4)
O1—Co1—N3—C12	−157.47 (18)	C9—C8—C10—C11	−177.9 (3)
O4—Co1—N3—C12	59.53 (19)	C8—C10—C11—C12	0.2 (4)
N1—Co1—N3—C12	−50.79 (19)	C7—N3—C12—C11	1.3 (4)
O3—Co1—N3—C12	114.65 (18)	Co1—N3—C12—C11	−174.1 (2)
O1—Co1—N3—C7	27.4 (2)	C10—C11—C12—N3	−1.4 (4)
O4—Co1—N3—C7	−115.6 (2)	Co1—O1—C13—O2	−4.2 (4)
N1—Co1—N3—C7	134.1 (2)	Co1—O1—C13—C14	175.0 (3)
O3—Co1—N3—C7	−60.5 (2)	Co1—O3—C15—O4	0.9 (3)
C6—N1—C1—N2	−179.0 (3)	Co1—O3—C15—C16	−179.2 (3)
Co1—N1—C1—N2	13.1 (4)	Co1—O4—C15—O3	−1.1 (3)
C6—N1—C1—C2	3.1 (4)	Co1—O4—C15—C16	179.0 (2)

### Hydrogen-bond geometry (Å, º)

**Table:**

*D*—H···*A*	*D*—H	H···*A*	*D*···*A*	*D*—H···*A*
N2—H2*A*···O4	0.85 (2)	2.17 (2)	2.965 (3)	157 (3)
N2—H2*B*···O2^i^	0.84 (2)	2.16 (2)	2.978 (3)	166 (3)
N4—H4*A*···O1	0.83 (3)	2.10 (3)	2.859 (3)	153 (3)
N4—H4*B*···O3^ii^	0.84 (2)	2.06 (2)	2.881 (3)	164 (3)

Symmetry codes: (i) −*x*+1, −*y*+2, −*z*+2; (ii) −*x*+2, −*y*+2, −*z*+1.
